# Cytotoxic mechanisms of berberine-phytantriol liquid crystalline nanoparticles against non-small-cell lung cancer

**DOI:** 10.17179/excli2023-6156

**Published:** 2023-06-19

**Authors:** Keshav Raj Paudel, Bikash Manandhar, Sachin Kumar Singh, Gaurav Gupta, Philip Michael Hansbro, Dinesh Kumar Chellappan, Kamal Dua

**Affiliations:** 1 Centre for Inflammation, Centenary Institute and University of Technology Sydney, Faculty of Science, School of Life Sciences, Sydney, NSW 2050, Australia; 2 Discipline of Pharmacy, Graduate School of Health, University of Technology Sydney, Sydney, NSW 2007, Australia; 3 Faculty of Health, Australian Research Centre in Complementary & Integrative Medicine, University of Technology Sydney, Ultimo, NSW 2007, Australia; 4 School of Pharmaceutical Sciences, Lovely Professional University, Phagwara, Punjab 144411, India; 5 School of Pharmacy, Suresh Gyan Vihar University, Jagatpura 302017, Mahal Road, Jaipur, India; 6 Center for Transdisciplinary Research, Saveetha Institute of Medical and Technical Science, Saveetha University, Chennai, India; 7 Uttaranchal Institute of Pharmaceutical Sciences, Uttaranchal University, Dehradun 248007, India; 8 Department of Life Sciences, School of Pharmacy, International Medical University, Bukit Jalil, Kuala Lumpur 57000, Malaysia

## ⁯

Lung cancer is the leading cause of cancer-related deaths worldwide, with 1.79 million reported deaths in the year 2020 alone (Thai et al., 2021[18]). Non-small-cell lung cancer (NSCLC) makes up ~85 % of total cancer cases, making it the most prevalent form of lung cancer. Tobacco smoking, environmental pollutants and genetic predisposition are the main factors that contribute to the pathogenesis of NSCLC. The therapeutic management of NSCLC and the disease progression include surgical resection, radiotherapy, chemotherapy, and immunotherapy. However, toxicity and safety issues of these strategies underpin the unmet need for the development of a targeted drug therapy with minimal adverse effects.

Several proteins play a critical role in the progression of NSCLC primarily by regulating tumor cell proliferation, growth, and apoptosis. Among them, survivin, hypoxia inducible factor (hif)-1α and p27^KIP1^ are considered important biomarker proteins in NSCLC. Survivin is an inhibitor of apoptosis and is overexpressed in most cancer types, including NSCLC (Jaiswal et al., 2015[8]). For example, survivin mRNA levels were elevated in 96 % of stage I surgically resected NSCLC from 83 patients. Clinical studies have shown that the level of survivin correlates with the overall survival of NSCLC patients who underwent surgery and is considered a prognostic factor in NSCLC (Fan et al., 2008[4]; Rosato et al., 2013[16]). Hif-1α is the regulatory subunit of hif-1 and acts as a key mediator of the cellular response to hypoxia, the most common feature of solid tumor progression, by regulating the genes associated with several physiological processes including cell metabolism, inhibition of apoptosis, tumor cell proliferation and metastasis (Harris, 2002[6]). The adaptive responses activated by hif-1α can make the tumor more aggressive by accelerating tumor progression, invasion, and metastasis. Like survivin, hif-1α levels are increased in several tumors, including NSCLC, and is associated with poor prognosis (Giatromanolaki et al., 2001[5]; Lau et al., 2007[10]). On the other hand, cyclin-dependent kinase inhibitor p27^KIP1^ is a putative tumor suppressor protein that negatively regulates cell cycle progression by binding to cyclin-cyclin dependent kinase complexes and inhibits their catalytic activity to induce cell-cycle arrest (Lloyd et al., 1999[12]). P27^KIP1^ is also known to modulate apoptosis (Katayose et al., 1997[9]). NSCLC shows low or undetectable levels of p27 and correlates with overall survival times of patients who underwent resection (Esposito et al., 1997[3]; Slingerland and Pagano, 2000[17]). 

Berberine is an isoquinoline alkaloid that is mostly found in the plant species of *Berberis*. Berberine containing plants have been traditionally used in the treatment of several pathologies including inflammation, diabetes, and infectious diseases (Neag et al., 2018[14]). Owing to its beneficial effects in several diseases including diabetes, cardiovascular diseases, and cancer, berberine is commercially available as dietary supplements. However, berberine has low bioavailability and is extremely toxic at higher doses, which limit its therapeutic benefit against NSCLC. 

The therapeutic approach of targeted killing of cancer cells has been widely studied for the treatment of NSCLC. As such, survivin, hif-1α and p27^KIP1^ are potential targets for developing effective anti-cancer therapy against NSCLC. Recently, a novel formulation of berberine-phytantriol-loaded liquid crystalline nanoparticles (BP-LCNs) reportedly demonstrated enhanced anti-cancer efficacy against NSCLC in an *in vitro* model of human lung adenocarcinoma A549 cells by inhibiting cell proliferation and metastasis (Alnuqaydan et al., 2022[1]). Therefore, in this study, the therapeutic roles of BP-LCNs on the regulation of survivin, hif-1α and p27^KIP1^ were evaluated in A549 cells.

BP-LCNs were optimally formulated as described previously (Alnuqaydan et al., 2022[1]). A549 human lung epithelial carcinoma cell line (ATCC, Manassas, VA, USA) was obtained as a kind gift from Prof. Alaina Ammit (Woolcock Institute of Medical Research, Sydney, Australia). A549 cells were cultured in low-glucose Dulbecco's modified Eagle's medium (DMEM, Lonza, Basel, Switzerland), supplemented with 5 % (*v*/*v*) fetal bovine serum (Lonza) and 1 % (*v*/*v*) penicillin and streptomycin mix (Lonza) in a humidified environment, maintained at 37 °C and 5 % CO_2_. The cells were seeded on 6-well plates at a density of 2×10^5^ cell/well. At 80 % confluency, the cells were incubated for 24 h at 37 ºC in the absence or presence of BP-LCNs (final concentration 5 µM). The cells were then washed 2× with PBS and lysed with RIPA buffer (Roche Diagnostics, Basel, Switzerland) and were subsequently stored at −80 °C until used further for protein array analysis. The changes in the protein expression levels of survivin, hif-1α and p27^KIP^ in A549 cells were determined using a Human XL oncology array kit (R&D Systems, Minneapolis, MN). Cell lysates (equivalent to 300 µg protein for each sample) were run on a Human XL oncology array following the manufacturer's protocol. The protein signals obtained in the array were imaged with ChemiDoc MP imaging system (Bio-Rad, Hercules, CA, USA). The pixel densities of the protein signals in the images were quantified using the Image J software. The data was analyzed by a two-tailed unpaired *t*-test using GraphPad Prism v.9.4.0. A p-value <0.05 was considered statistically significant.

We observed that the protein levels of survivin and hif-1α in A549 cells incubated with BP-LCNs were downregulated by 60.9 % (supplementary information, Figure 1A, p<0.01 versus control) and 48.2 % (Figure 1B, p<0.05 versus control), respectively, as compared to the control. On the other hand, the protein level of p27^KIP1^ in A549 cells incubated with BP-LCNs was upregulated by 91.6 % (Figure 1C, p<0.01 versus control), compared to the control. The findings of this study complement the evidence of mechanism demonstrating cytotoxic activity of BP-LCN formulation against NSCLC (Alnuqaydan et al., 2022[1]). This study showed that BP-LCNs may regulate the expression of important biomarkers of NSCLC such as survivin, hif-1α and p27^KIP1^. BP-LCNs significantly downregulated the protein expression of survivin and hif-1α that are associated with tumor growth and metastasis, and upregulated the protein expression of p27^KIP1^, a modulator of apoptosis, in A549 cells. These findings signify the potential of BP-LCN formulation for translation into clinical settings as a therapeutic dosage regimen against NSCLC.

In an *in vitro* study, downregulation of survivin expression by knockdown of survivin gene has been shown to decrease cell proliferation, inhibit colony formation and induce apoptosis in A549 cells (Zhang et al., 2015[20]). Survivin expression also correlates inversely with the expression of tumor suppressor protein p53 and positively with tumor proliferation (Ulukus et al., 2007[19]). Hence, the downregulation of survivin by BP-LCNs could be one of the major contributors of the anti-proliferative and cytotoxic effects of BP-LCNs as observed in the previous study (Alnuqaydan et al., 2022[1]), where p53 was found to be upregulated by BP-LCNs. Expression of p27^KIP1^ has been shown to induce cell death in A549 cells (Ishii et al., 2004[7]). The observed increase in p27^KIP1^ in BP-LCN-treated A549 cells also supports the BP-LCN-mediated decrease in A549 cell proliferation and colony formation as observed previously (Alnuqaydan et al., 2022[1]). In addition to tumor growth and invasiveness, increased hif-1α expression is associated with decreased sensitivity to chemotherapy and radiation therapy (Rankin and Giaccia, 2008[15]). Hence, treatment with BP-LCNs may increase the sensitivity of NSCLC to chemotherapy and radiotherapy by decreasing the hif-1α expression. 

Various therapeutic drug molecules have been studied to evaluate their efficacies against different types of cancer including NSCLC by modulating the expression of survivin, hif-1α and p27^KIP1^ (Choi et al., 2009[2]; Li et al., 2019[11]; Naruse et al., 2000[13]). Based on the findings from this study, BP-LCN formulation presents as a promising therapeutic candidate against NSCLC by regulating apoptosis and cell proliferation, associated with the functions of survivin, hif-1α and p27^KIP1^.

This study provides evidence for the anti-cancer potential of BP-LCN formulation against NSCLC by decreasing the levels of survivin and hif-1α and increasing the levels of p27^KIP1^. These findings support the potential of future development of BP-LCNs as therapeutic drug regimen for the treatment against NSCLC.

## Notes

Dinesh Kumar Chellappan and Kamal Dua (Discipline of Pharmacy, Graduate School of Health, University of Technology Sydney, Sydney, NSW 2007, Australia; E-mail: Kamal.Dua@uts.edu.au) contributed equally as corresponding author.

## Conflict of interest

The authors declare that they have no conflict of interest.

## Supplementary Material

Supplementary information
