# Supplementary material for: Cytotoxic mechanisms of berberine-phytantriol liquid crystalline nanoparticles against non-small-cell lung cancer
Source: EXCLI J. 2023 Jun 19;22:516–9. doi: 10.17179/excli2023-6156 (PMC10390893; doi:10.17179/excli2023-6156)
Supplement: Supplementary information [file EXCLI-22-516-s-001.pdf]

EXCLI Journal 2023;22:516-519 – ISSN 1611-2156
Received: May 06, 2023, accepted: June 15, 2023, published: June 19, 2023

Supplementary information to:

Letter to the editor:

CYTOTOXIC MECHANISMS OF BERBERINE-PHYTANTRIOL LIQUID CRYSTALLINE NANOPARTICLES AGAINST NON-SMALL-CELL LUNG CANCER

Keshav Raj Paudel[1], Bikash Manandhar[2,3], Sachin Kumar Singh[3,4], Gaurav Gupta[5,6,7], Philip Michael Hansbro[1], Dinesh Kumar Chellappan[8,*], Kamal Dua[2,3,*]

[1] Centre for Inflammation, Centenary Institute and University of Technology Sydney, Faculty of Science, School of Life Sciences, Sydney, NSW 2050, Australia
[2] Discipline of Pharmacy, Graduate School of Health, University of Technology Sydney, Sydney, NSW 2007, Australia
[3] Faculty of Health, Australian Research Centre in Complementary & Integrative Medicine, University of Technology Sydney, Ultimo, NSW 2007, Australia
[4] School of Pharmaceutical Sciences, Lovely Professional University, Phagwara, Punjab 144411, India
[5] School of Pharmacy, Suresh Gyan Vihar University, Jagatpura 302017, Mahal Road, Jaipur, India
[6] Center for Transdisciplinary Research, Saveetha Institute of Medical and Technical Science, Saveetha University, Chennai, India
[7] Uttaranchal Institute of Pharmaceutical Sciences, Uttaranchal University, Dehradun 248007, India
[8] Department of Life Sciences, School of Pharmacy, International Medical University, Bukit Jalil, Kuala Lumpur 57000, Malaysia

* **Corresponding authors:** Dr. Kamal Dua, Discipline of Pharmacy, Graduate School of Health, University of Technology Sydney, Sydney, NSW 2007, Australia.
E-mail: Kamal.Dua@uts.edu.au
Dr. Dinesh Kumar Chellappan, Department of Life Sciences, School of Pharmacy, International Medical University, Bukit Jalil, Kuala Lumpur 57000, Malaysia.
E-mail: Dinesh_Kumar@imu.edu.my

https://dx.doi.org/10.17179/excli2023-6156

This is an Open Access article distributed under the terms of the Creative Commons Attribution License (http://creativecommons.org/licenses/by/4.0/).

EXCLI Journal 2023;22:516-519 – ISSN 1611-2156
Received: May 06, 2023, accepted: June 15, 2023, published: June 19, 2023

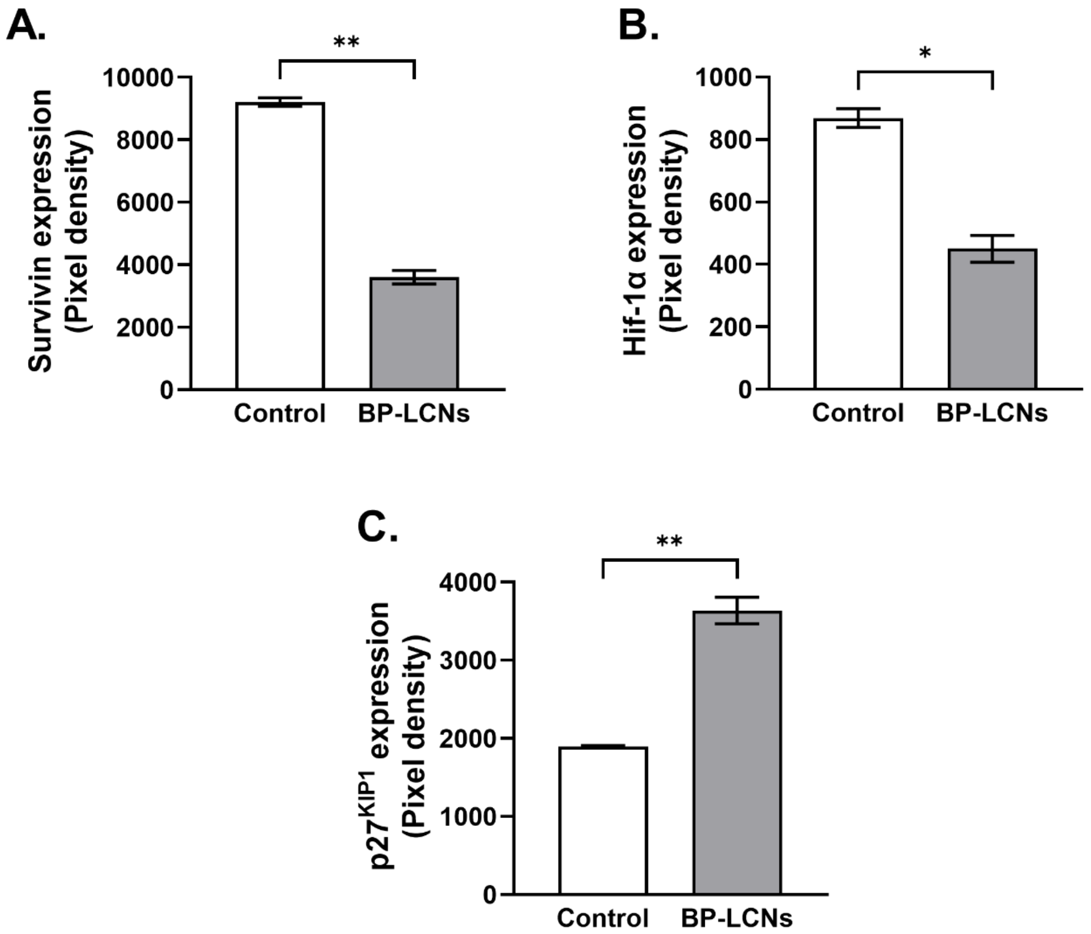

Figure 1: Effect of BP-LCNs on the protein levels of survivin, hif-1α and p27^{KIP1}. A549 cells were incubated at 37 ºC for 24 h in the absence or presence of BP-LCNs (final concentration 5 µM). The cells were lysed and the protein expressions of survivin (**Panel A**), hif-1α (**Panel B**) and p27^{KIP1} (**Panel C**) were determined with human XL oncology array kit. The values represent mean ± SEM of three independent experiments.
*p<0.05; **p<0.01
